# Search for Limiting Factors in the RNAi Pathway in Silkmoth Tissues and the Bm5 Cell Line: The RNA-Binding Proteins R2D2 and Translin

**DOI:** 10.1371/journal.pone.0020250

**Published:** 2011-05-26

**Authors:** Luc Swevers, Jisheng Liu, Hanneke Huvenne, Guy Smagghe

**Affiliations:** 1 Insect Molecular Genetics and Biotechnology, Institute of Biology, National Centre for Scientific Research “Demokritos,” Athens, Greece; 2 Laboratory of Agrozoology, Department of Crop Protection, Faculty of Bioscience Engineering, Ghent University, Ghent, Belgium; French National Center for Scientific Research - Institut de biologie moléculaire et cellulaire, France

## Abstract

RNA interference (RNAi), an RNA-dependent gene silencing process that is initiated by double-stranded RNA (dsRNA) molecules, has been applied with variable success in lepidopteran insects, in contrast to the high efficiency achieved in the coleopteran *Tribolium castaneum*. To gain insight into the factors that determine the efficiency of RNAi, a survey was carried out to check the expression of factors that constitute the machinery of the small interfering RNA (siRNA) and microRNA (miRNA) pathways in different tissues and stages of the silkmoth, *Bombyx mori*. It was found that the dsRNA-binding protein R2D2, an essential component in the siRNA pathway in *Drosophila*, was expressed at minimal levels in silkmoth tissues. The silkmoth-derived Bm5 cell line was also deficient in expression of mRNA encoding full-length BmTranslin, an RNA-binding factor that has been shown to stimulate the efficiency of RNAi. However, despite the lack of expression of the RNA-binding proteins, silencing of a luciferase reporter gene was observed by co-transfection of *luc* dsRNA using a lipophilic reagent. In contrast, gene silencing was not detected when the cells were soaked in culture medium supplemented with dsRNA. The introduction of an expression construct for *Tribolium* R2D2 (TcR2D2) did not influence the potency of *luc* dsRNA to silence the luciferase reporter. Immunostaining experiments further showed that both TcR2D2 and BmTranslin accumulated at defined locations within the cytoplasm of transfected cells. Our results offer a first evaluation of the expression of the RNAi machinery in silkmoth tissues and Bm5 cells and provide evidence for a functional RNAi response to intracellular dsRNA in the absence of R2D2 and Translin. The failure of TcR2D2 to stimulate the intracellular RNAi pathway in *Bombyx* cells is discussed.

## Introduction

RNA interference (RNAi) is the cellular process of sequence-specific gene silencing in response to the presence of homologous dsRNA. Its discovery heralded a revolution in biology, because suddenly a technique was available that allowed to analyze gene function rapidly and that was not restricted to model organisms for which extensive genetic tools were developed, such as *Drosophila melanogaster* [1]–[4]. RNAi is involved in a diverse set of gene-regulatory mechanisms, including the silencing of endogenous genes during development and defense against virus infection and spread of endogenous mobile repetitive DNA sequences in the genome [5]. A hallmark of RNAi is the generation of small (20–30 nt) RNAs that function as specificity factors in the silencing process. Currently, three classes of small RNAs have been identified. The first two classes, small interfering RNAs (siRNAs) and microRNAs (miRNAs), are generated by processing of long precursor dsRNAs into small RNA duplexes by RNaseIII-type Dicer enzymes [5], [6]. The RNA duplexes are subsequently unwound and loaded into large RNA-induced silencing complexes (RISCs). RISC complexes actively search mRNAs for complementary target sequences and initiate silencing by endonuclease cleavage (siRNAs) or translation inhibition (miRNAs) [7]. More recently, a third class of small RNAs, PIWI-associated RNAs or piRNAs, was discovered. These small RNAs belong to a different size class, are generated independent of Dicer activity, and are proposed to be primarily involved in suppression of mobile genetic elements in the germline [8]. In this article, the focus is on siRNAs and miRNAs since these small RNAs are considered predominant in somatic cells.

For *in vivo* applications in insects, the most convenient way to achieve gene silencing would be by injection of dsRNA into the hemolymph of the insect. This approach, in which silencing is achieved in different cells or tissues after internalization of dsRNA, is called “systemic RNAi” and has led to successful knockdown of the target gene in a considerable number of reports [3], [4], [9]. While the technique of systemic RNAi is very efficient in coleopteran insects such as *Tribolium castaneum* [10], a much smaller rate of efficiency has been reported for lepidopteran insects [11]. In the silkmoth *Bombyx mori*, an important model for lepidopteran insects, a large variety in RNAi efficiency has been reported: while studies targeting genes that regulate molt and metamorphosis were largely unsuccessful, in other studies that targeted the immune system, the silk gland and the pheromone gland, efficient gene silencing was achieved. These data indicate that tissue-specific factors may be involved (database of RNAi studies at http://insectacentral.org/RNAi; [11]). However, the identities of these factors that determine the efficiency of RNAi in *Bombyx* and other Lepidoptera remain unknown.

*Drosophila* embryo lysates and extracts from *Drosophila*-derived S2 cells have been used to elucidate the mechanism of RNA silencing [12] and can serve as model for the functioning of the RNAi machinery in other insects. In *Drosophila*, the two RNA silencing pathways, that involve siRNAs and miRNAs, respectively, seem to be separated with respect to their biogenesis and function. Both types of small RNAs are generated by separate Dicer enzymes (nuclear Drosha and cytoplasmic Dicer-1 for miRNAs and Dicer-2 for siRNAs) and enter RISC assembly pathways with separate Argonaute proteins as core constituents (Ago-1 for miRNAs and Ago-2 for siRNAs) [13]. The Dicer enzymes generally also function as a complex with dsRNA-binding proteins that are specific to each pathway (Drosha/Pasha and Dicer-1/Loquacious for miRNA pathway and Dicer-2/R2D2 for the siRNA pathway [12], [13]). In general, it is thought that the miRNA pathway primarily uses endogenous products from the cell's genome with dsRNA structure (transcribed from endogenous genes as stem-loop precursors) to regulate developmental processes, while the siRNA pathway is a defense response against exogenous dsRNAs, as for instance generated from viruses. More recently, it was recognized that endogenous long inverted repeat transcripts with full complementarity enter a “hybrid” RNA silencing pathway that is dependent on Dicer-2 and Ago-2 as expected, but also involves the Dicer-1-cofactor Loquacious [14]. This finding illustrates that siRNA and miRNA pathways can be partially overlapping in *Drosophila*.

Besides the above mentioned *in vitro* systems derived from *Drosophila*, nothing is known regarding the functioning of the RNAi machinery in other insects, including *Tribolium* which is known for its sensitivity to RNAi. In the case for lepidopteran insects, such knowledge could be valuable, as it could lead to the development of new methods to increase RNAi efficiency in this order. To gain insight in the mechanism of the RNAi process in lepidopteran insects, we have carried out gene expression studies of the major factors of the siRNA and miRNA pathways in different tissues and at different stages in the silkmoth. In addition, we have focused on the silkmoth-derived Bm5 cell line which is derived from ovarian tissue [15]. This cell line can be easily transfected and transformed [16] and therefore genetically manipulated to test functions of individual factors in small RNA signalling pathways. A key finding was the absence of expression of R2D2, an auxiliary factor that is essential for Dicer-2 function in siRNA-mediated RNAi in *Drosophila* S2 cells [17]. Bm5 cells were also deficient in expression of functional BmTranslin protein, another RNAi regulator identified in *Drosophila* S2 cells [18]. Despite the absence of R2D2 and Translin, however, Bm5 cells were capable of specific gene silencing when dsRNA was transfected into the cells. In rescue experiments, no increase in potency of dsRNA to trigger RNAi was observed when *Tribolium* R2D2 (TcR2D2) was co-expressed. This could indicate that RNAi efficiency in silkmoth cells is not influenced by the R2D2 factor or could reflect the inability of TcR2D2 to enter the siRNA pathway in Bm5 cells. Immunostaining experiments indicate that TcR2D2 and BmTranslin are located at defined locations in the cytoplasm of culture cells, and likely play a role in RNA (for instance, mRNA or miRNA) localization associated with specific subcellular functions.

## Results

### Expression studies of key factors of RNA silencing pathways

In lepidopteran insects, different silencing efficiencies by dsRNAs are observed between different tissues [11]. As a first approach to provide an explanation for these differences, we investigated the expression of the mRNAs that encode factors of the core machinery of the siRNA and miRNA silencing pathways. In this first series of experiments, the presence/absence of the mRNAs was investigated after 35 cycles in RT-PCR, without investigating in detail by semi-quantitative or quantitative (real-time) RT-PCR experiments absolute or relative differences in quantity of transcripts among different tissues.

**Figure 1:**
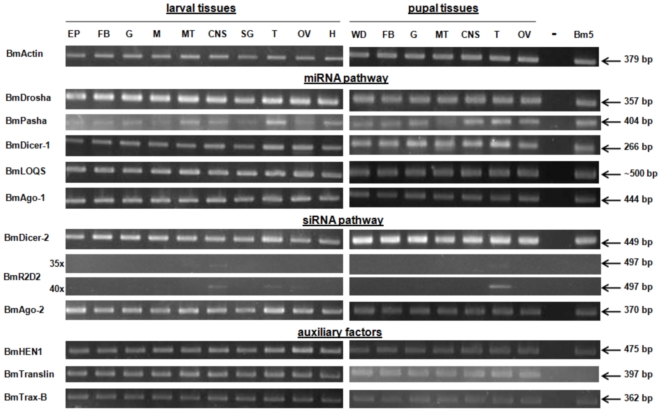
Amplification of cDNAs of factors of the core machinery of small RNA pathways from different larval and pupal tissues from the silkmoth strain Daizo and the silkmoth-derived Bm5 cell line using primers described in **Table 1**. The products that are shown were obtained after 35 cycles of PCR, with the exceptions of actin cDNA, which was amplified after 30 cycles, and BmR2D2 which was amplified for both 35 and 40 cycles. Note the very low to absent of expression of BmR2D2 in all samples and the absence of expression of BmTranslin in Bm5 cells. Indicated at the right is the size of the amplified product. PCR products from selected tissues were sequenced and shown to correspond to the cDNA (spanning different exons in the genome) of the indicated gene (**Text S1**). For BmR2D2, the results are shown for primer pair 1 (**Table 1**), using DNase-treated RNA as template for the RT-PCR reactions. For BmTranslin, the reaction product obtained with primer pair 1 is shown, which amplifies specifically the BmTranslin2 isoform. Abbreviations: larval tissues: EP = epidermis; FB = fat body; G = midgut; M = muscle; MT = Malpighian tubules; CNS = central nervous system; SG = silk gland; T = testis; OV = ovary; H = haemocytes; pupal tissues: WD = wing disk; FB = fat body; G = midgut; MT = Malpighian tubules; CNS = central nervous system; T = testis; OV = ovary. The column “-” shows amplifications in the absence of template with the exception of the reaction with BmR2D2 primers that shows the fragment amplified from genomic DNA.

As is evident from **Figure 1**, almost all factors involved in the small RNA silencing pathways have a very broad expression pattern of their mRNAs. Only the mRNA expression of the *Bombyx* Pasha homolog showed clear differences among different tissues, while differences in expression of other factors are generally much less obvious. Of interest is the very low to absent expression of *Bombyx* R2D2 (BmR2D2) mRNA, despite the presence of the gene in the *Bombyx* genome and the annotation of its mRNA in Genbank (accession number NM_001195078).

The detection of BmR2D2 mRNA was attempted using different sets of primers (**Table 1**). Using primer pair 1, PCR products of different sizes were detected. In one series of amplifications, a fragment of the corrected size (497 bp) was generated from nervous system and gonads of larvae and from testis tissue of pupae (**Figure 1**). Clear amplification of this product was only observed after a PCR of 40 cycles (**Figure 1**). In a second series of amplifications, however, carried out on a independent set of cDNAs, a truncated product of 414 bp was generated from pupal testis tissue while no clear amplification products became apparent from other tissues after 40 cycles of PCR (**Figure S1**). After sequencing of this product, differences at the 3^rd^ exon were revealed when comparing with the cDNA sequence in Genbank and the 497 bp PCR product from the first set of independent cDNA samples (**Figure S1**) (see also below).

**Table 1:** Primer pairs used for amplification of factors of the core machinery of small RNA pathways.

Gene	Forward Primer	Reverse Primer
**miRNA pathway**		
BmDrosha	CGTTCACGGATCGCTCAGTC	GCGGAAACAGACACGCGTTG
BmPasha	GTCAGCACCAGCTGGAAGAC	ACTGCATGCCGCCGATGTAC
BmDicer-1	TGAAGCCGGGTGAGGTGTTC	GGGAGTGAGGAGGTTGAGTC
BmLoquacious	GAGCTGTTGGCACGTCGTGG	GTTGTCCTGTAAGGCCTGCC
BmAgo-1	GGGCGATAGCATGTTTCGCG	TCACGTCCACGCCCAGGAAG
**siRNA pathway**		
BmDicer-2	CATACAGTTCACCGAAGAGG	GGATGTACGACGAGTGAGAC
BmR2D2 (1)	CAAGATGAAAACTCCCATAACAGTACTG	TTTGTCGCGCCTGTCGCTTG
BmR2D2 (2)	CAAGATGAAAACTCCCATAACAGTACTG	TCACAGAGCGGCGGGCGGCGGA
BmR2D2 (3)	AAAGCGCCCACAGTGGACAG	TCACAGAGCGGCGGGCGGCGGA
BmR2D2 (4)	CCGCTGTAAGGCTTTAGGTGAG	TCACAGAGCGGCGGGCGGCGGA
BmR2D2 (5)	AAAGCGCCCACAGTGGACAG	TTTGTCGCGCCTGTCGCTTG
BmAgo-2	TCTCCGATTGACTTGGGCGAC	ATACGGTCATCCTAACCGGCG
**common factors**		
BmHen1	CCCACCAATGTATGTACAGC	ATGATCGAGCCTTCTCAAGC
BmTranslin (1)	AGGTATCAGGATCACTGGAG	TGATGCTGAGATCGTAGACG
BmTranslin (2)	AAAAATGTGCGATAATGAATTGATC	CCTCTATTTTCTCTTCAAGTTCAATAG
BmTranslin (3)	AAAAATGTGCGATAATGAATTGATC	TTTATTATTTATGTTCGTGTTCTGGTCC
BmTrax-B	ACAGTCCGATCTTAGCCATG	TTCATCTCGTATCTCTGGCC

All primers amplified specific PCR products (**Figure 1**; sequences shown in **Text S1**) with exception of some BmR2D2 primer pairs as discussed in the text. BmR2D2 primer pair 1 amplified the cDNA fragments that are shown in **Figure 1** and **Figure S1A**. BmR2D2 primer pair 5 amplified a 196 bp fragment from genomic DNA (**Figure S1C** and **Text S1**) but not from any cDNA. The use of BmR2D2 primer pairs 2, 3 and 4 did not result in any specific amplification products either from cDNA or from genomic DNA. BmTranslin primer pair 1 detects specifically the BmTranslin2 isoform but not the BmTranslin1 isoform (amplified product shown in **Figure 1**; sequence shown in **Text S1**). BmTranslin primer pairs 2 and 3 were used to amplify the complete ORF of the BmTranslin1 and BmTranslin2 isoforms, respectively.

Other primer pairs that were designed to amplify other parts of BmR2D2 mRNA, were unsuccessful (**Table 1**). It should also be noted that primer pair 5, that amplifies a sequence within the third exon, was successful using genomic DNA as template, but not using cDNA derived from DNase-treated RNA (**Figure S1**), indicating functionality of the relevant primer pair. Moreover, PCR fragments from primers that cover the 3′-part of the R2D2 gene for 35–40 cycles, were shown to be non-specific after sequencing. Therefore, it is concluded that the BmR2D2 mRNA is either not expressed or only expressed at very low levels in the tissues of the *Bombyx* Daizo strain that was investigated.

**Figure 2:**
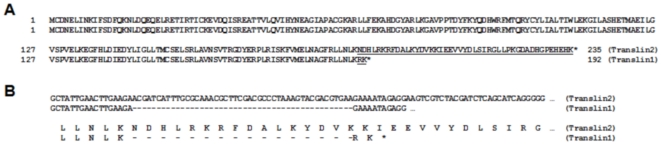
The sequences of BmTranslin isoforms. **A.** Amino-acid sequences of the two BmTranslin isoforms isolated from silkmoth tissues. The protein isoforms have different C-terminal ends (underlined). **B.** Comparison of the BmTranslin isoform sequences which shows the deletion in the BmTranslin1 sequence that causes a frameshift and a premature stop codon.

The expression pattern of factors of the small RNA pathways in Bm5 cells followed the general trend present in the silkmoth tissues (and includes absence of BmR2D2 mRNA; **Figure 1**), with the exception of the amplification of BmTranslin mRNA using primer pair 1 (**Table 1**; this primer pair is specific to the Genbank sequence NM_001046817; corresponding to “BmTranslin2”, see below) that did not result in a specific PCR product for the cell line. However, an alternative isoform of BmTranslin mRNA, with a truncation of the ORF at the C-terminus, “BmTranslin1”, was detected in both silkmoth tissues and Bm5 cells using primer pair 2 (**Table 1**). An EST of this isoform was also present in Silkbase (fdpeP14_F_N15.2). **Figure 2A** represents the complete amino-acid sequences of both the truncated isoform (“BmTranslin1”, amplified by primer pair 2 (**Table 1**), corresponding to EST fdpeP14_F_N15.2, and expressed in both silkmoth tissues and Bm5 cells) and the extended isoform (“BmTranslin2”, amplified by primer pair 3 (**Table 1**), corresponding to Genbank accession NM_001046817, and expressed in silkmoth tissues but not in Bm5 cells). It is noted that the conserved C-terminus present in Translin proteins in general [19] is not present in the BmTranslin1 isoform.

### Evidence for presence of mutant BmR2D2 and BmTranslin genes

When the amplified fragment of 414 bp corresponding to the 5′-part of BmR2D2 mRNA (amplified from testis tissue in the second set of amplifications by primer pair 1, see above) was compared to the genome sequence of *Bombyx*, differences in the sequence of the third exon were revealed (**Figure S1**). It was observed that a sequence of 83 nt was deleted from the sequence of the PCR fragment. Because this sequence is not flanked by consensus intron splice recognition sequences (GT … AG), the most likely explanation must be that the exon has undergone a small deletion and therefore corresponds to a mutant allele of the *BmR2D2* gene.

Similarly, comparing the sequences of the cDNAs of BmTranslin1 and BmTranslin2 with the genome sequence, it is noted that, in the BmTranslin1 sequence, 46 nt are deleted from the exon that encodes the C-terminus of BmTranslin (**Figure 2B**). As for the *BmR2D2* gene, the deleted sequence is not bracketed by consensus intron splice sites and therefore may correspond to a mutant allele of *BmTranslin*.

It is noted that the changes in the mutant alleles of *BmR2D2* and *BmTranslin* result in frameshifts for both genes and in a premature stop codon for *BmTranslin1*. This could indicate that the function of the mutant proteins is significantly altered.

### The intracellular RNAi response in Bm5 cells and the lack of stimulation by *Tribolium* R2D2

Using tissue culture cells, two types of RNAi can be investigated, depending on the method of dsRNA administration. In case of intracellular RNAi, dsRNA is introduced into the cells with high efficiency by transfection with a lipophilic reagent and the RNAi response can be thought to depend only on the competence of the core RNAi machinery in the cell. However, in some cell lines, such as *Drosophila* S2 cells, it is observed that simple “soaking” of the cells in growth medium supplemented with dsRNA at concentrations between 5–20 µg/ml can result in silencing effects [20], [21]. In such experiments, it was observed that dsRNA is internalized by the cells through the endocytosis pathway [20] prior to its presentation to the intracellular RNAi machinery.

**Figure 3:**
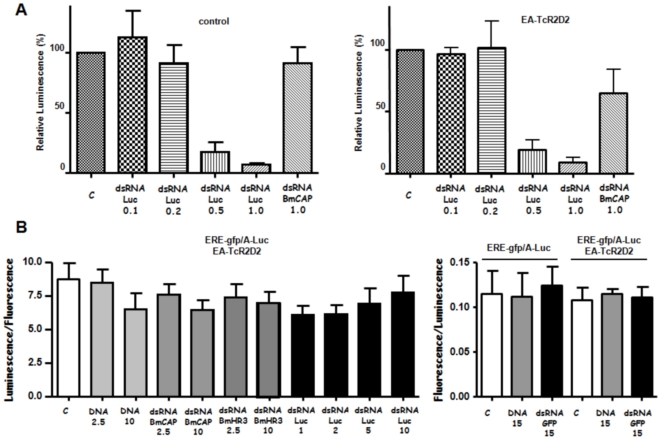
RNAi functional assays. **A.** Functional assay after transfection of dsRNA. Bm5 cells were transfected with DNA and either non-specific dsRNA or specific dsRNA at different ratios in the absence (**Left**) or presence (**Right**) of TcR2D2 expression plasmid. Indicated is the amount of luminescence in comparison with cells that received an equivalent amount of non-specific DNA (C; 100%). Experimental conditions include treatment with BmCAP-dsRNA and Luc-dsRNA at the indicated amounts (µg/ml in transfection mixture). N = 3. **B.** Functional assay after addition of nucleic acid (DNA or dsRNA) to the tissue culture medium. Cells were soaked with extracellular dsRNA for two days before transfection and for three days after transfection. Experimental conditions include treatment with DNA, BmCAP-dsRNA, BmHR3-dsRNA, Luc-dsRNA and GFP-dsRNA at the indicated concentrations (in µg/ml). **Left:** Luciferase assays to evaluate expression of the constitutive A-Luc transgene. Indicated is the luminescence/fluorescence ratio (N = 3). **Right.** Relative fluorescence measurements (fluorescence/luminescence ratios) 24 hrs after induction of the ecdysone ERE-gfp reporter with 200 nM of ecdysone agonist. Cells were soaked with nucleic acid (DNA or dsRNA) for two days before transfection and for another three days after transfection. At two days after transfection, cells were induced with hormone agonist for a period of 24 hours (N = 3).

It was observed that intracellular RNAi occurs efficiently in Bm5 cells in the absence of BmR2D2 expression. Co-transfection of the actin-luciferase reporter with Luc-dsRNA results in a dose-dependent reduction of luciferase activity (**Figure 3A**, left panel). This response is specific, since a high dose of a non-specific dsRNA (BmCAP-dsRNA) did not result in a decrease in luciferase activity. On the other hand, soaking of Bm5 cells with dsRNA in the extracellular medium at high concentration did not result in gene silencing (**Figure 3B**, right panel) as was observed before [22].

In *Drosophila*, genetic analysis has shown that the R2D2 protein is an important factor in the RNAi process through its role in the transfer of siRNAs to the Ago-2 effector protein [17], [23]. In *Drosophila*-derived S2 cells, it was shown that the Dicer-2/R2D2 complex is the principal siRNA-generating enzyme responsible for RNAi [17]. Because of its relative importance in the process of RNAi, at least in *Drosophila*, it was decided to focus on R2D2 to see whether its expression in Bm5 cells could stimulate the RNAi process in Bm5 cells.

**Figure 4:**
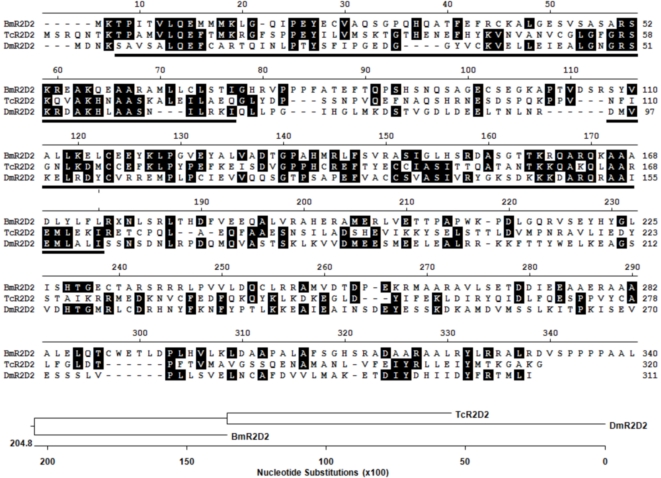
Alignment of the amino-acid sequences of the R2D2 proteins from *Bombyx mori* (BmR2D2), *Tribolium castaneum* (TcR2D2) and *Drosophila melanogaster* (DmR2D2) and the phylogenetic tree derived from it. Identical amino-acids are boxed in black. The two dsRNA-binding domains are underlined.

Because our unsuccessful attempts (see above) to amplify the ORF of *Bombyx* R2D2 from tissues of the silkmoth strain that is cultured in our laboratory (Daizo), it was decided to express the R2D2 homolog of *Tribolium castaneum* (TcR2D2; accession NM_001134953). The process of systemic RNAi, achieved through injection of dsRNA into the haemocoel, is very effective in this species. Therefore, *Tribolium* has become a major model to study the role of genes in developmental processes of molting and metamorphosis by RNAi-mediated gene knockdown [24], [25]. *TcR2D2* mRNA is abundant in *Tribolium* tissues (J. Baert, L. S., H. H., and G. S., unpublished results) and its high levels of expression are therefore correlated with the high efficiency of RNAi in this species. Although sequence conservation among R2D2 proteins of different species is low, TcR2D2 has the same domain structure as BmR2D2 (**Figure 4**) and may therefore enter the RNAi pathway in silkmoth cells (see also discussion).

The complete ORF of TcR2D2 was amplified from cDNA of *Tribolium* tissues, cloned in the pEA-MycHis lepidopteran expression vector and used in co-transfection experiments with luciferase reporter plasmid and dsRNA. However, it was observed that co-expression of TcR2D2 does not stimulate intracellular RNAi after transfection of dsRNA. As is shown in **Figure 3A**, right panel, the dose-dependent decrease in luciferase activity was not altered during co-transfection with TcR2D2 expression plasmid. In contrast to control transfections, in the presence of TcR2D2 a small knockdown (∼30%) was observed using non-specific dsRNA (**Figure 3A**, right panel). However, statistical analysis showed that the knockdown was not statistically significant (two-tailed student's *t*-test; P>0.05; N = 3).

### Absence of silencing in Bm5 cells after soaking with extracellular dsRNA and the lack of stimulation by *Tribolium* R2D2

One possible explanation for the deficiency in Bm5 cells to respond to dsRNA in the extracellular medium could be the inability of its core machinery apparatus (lacking BmR2D2) to become activated by the small amounts of dsRNA that are transported from the extracellular medium to the cytoplasm. It was therefore investigated whether ectopic expression of R2D2 protein is capable to sensitize the core RNAi machinery to the internalization of dsRNA and make the cell line competent to respond to extracellular dsRNA.

To test whether expression of TcR2D2 results in gene silencing following soaking with dsRNA, Bm5 cells were grown in culture medium containing Luc-dsRNA (or non-specific DNA or non-specific dsRNA) at different concentrations up to 10 µg/ml, a concentration sufficient to achieve efficient gene-knockdown in *Drosophila* S2 cells [26]. The cells were subsequently transfected with a luciferase reporter together with the TcR2D2 expression construct (as well as the ERE-gfp reporter which was used for normalization). Transfected cells were then again ‘soaked’ with dsRNA for an additional period of three days before evaluation. However, ectopic expression of TcR2D2 did not result in silencing of luciferase activity following addition of Luc-dsRNA to the culture medium for a total period of 5 days (**Figure 3B**, left panel).

It has also been reported that RNAi can work more efficiently on induced genes versus constitutive genes, when dsRNA is added prior to the induction [11]. To investigate this possibility, GFP-dsRNA was added to Bm5 cells before and after transfection with an ERE-gfp reporter construct in the absence or presence of the TcR2D2 expression construct. After induction of the ecdysone reporter by 200 nM ecdysone agonist also in this case no significant gene knockdown was observed (**Figure 3B**, right panel).

### Accumulation of TcR2D2 and BmTranslin2 at defined locations in the cytoplasm of tissue culture cells

**Figure 5:**
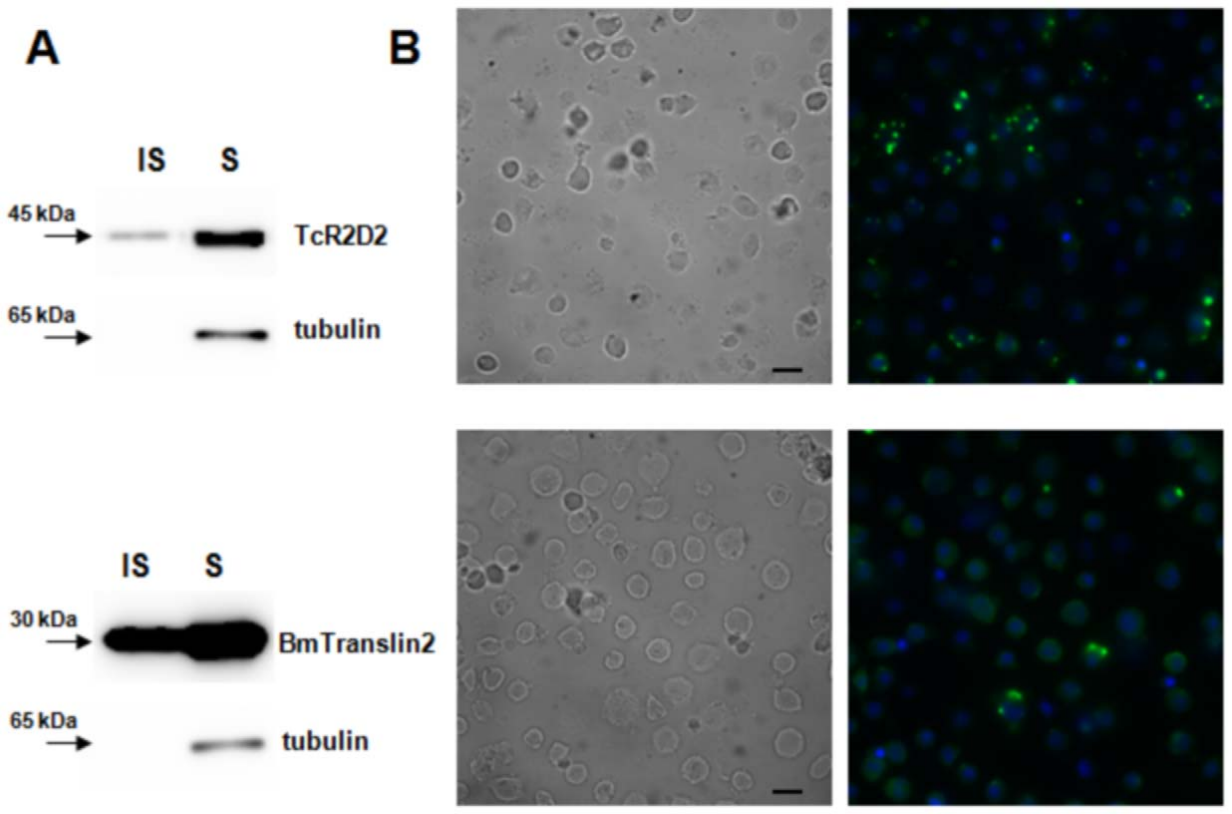
Expression of myc-tagged TcR2D2 (Upper) and BmTranslin2 (Lower) in transfected Hi5 cells. **Panel A:** Western blot analysis of expression of TcR2D2 and BmTranslin2. Both proteins accumulate preferentially in the soluble fraction (S) of the cell extracts compared to the insoluble fraction (IS). Exposure times are 3 minutes for BmTranslin2 and TcR2D2 and 2 min for tubulin. **Panel B:** Subcellular localization of myc-tagged TcR2D2 and BmTranslin2 in transfected Hi5 cells compared to DAPI (**Right**). Corresponding bright field images are shown at the **Left**. Scale bar in **Left Panel**: 15 µm.

Because high protein expression levels can be obtained in Hi5 cells following transfection [27], it was decided to use this cell line for the study of the subcellular localization of TcR2D2 and BmTranslin2. Following transfection of the expression constructs for both RNA-binding proteins, Western blot analysis indeed showed very high levels of expression of BmTranslin2 and high levels of TcR2D2 (**Figure 5A**). On the other hand, employment of expression constructs for BmTranslin1 resulted only in very low expression levels (**Figure S2**). Because the same type of expression vector was used in all cases, these results suggest that BmTranslin1 protein is unstable in Hi5 cells.

Immunofluorescence shows that both TcR2D2 and BmTranslin2 are located at defined positions in the cytoplasm of Hi5 cells (**Figure 5B**). However, the spot-like pattern of staining was more pronounced for TcR2D2 than for BmTranslin2. In the case of BmTranslin2, the majority of cells showed diffuse staining in the cytoplasm (**Figure 5B**). Quantitative analysis revealed that, of cells that were stained for TcR2D2, approximately 40% showed one spot, 29% displayed two spots while another 25% showed three or more spots (N = 129). While cells stained for BmTranslin2 mostly showed a diffuse pattern of staining (47%), approximately 22% showed one spot, 13% two spots while in the remaining 12% more than three spots were detected (N = 85). In a minority of cells, a crescent-type of staining was observed that was localized at one side of the cell (6% for both proteins).

## Discussion

In this study, a first attempt was undertaken to understand which factors may be limiting to RNAi in lepidopteran insects. In Lepidoptera, large differences in RNAi efficiency are observed among different species, developmental stages and even target genes [11]. It is understood that efficiency of RNAi can be influenced by many parameters, for instance expression levels of components of the core RNAi machinery, competence for uptake of dsRNA from the hemolymph or the gut content, and presence of dsRNA degrading enzymes in tissues. Our first approach to gain insight in this complex phenomenon focused on the expression of components of the RNAi machinery in different tissues and at different stages of the insect. Studies were carried out using the silkmoth *Bombyx mori* because of the availability of its genome sequence [28]–[30] that allows easy identification and subsequent detection of expression of genes that encode factors of the core machinery of the siRNA and miRNA pathways [5], [6]. Factors of the different pathways were looked at because it has been reported that different pathways overlap and can compete with each other [13], [31]. Moreover, it was reasoned that high expression of factors of the miRNA pathway (primarily involved in development) might interfere with the efficient operation of the siRNA pathway (primarily involved in viral and genome defense).

A major conclusion of this survey is that the majority of genes of the core small RNA machinery are ubiquitously expressed, with only minor differences among different tissues (**Figure 1**). Only *BmPasha*, which encodes a co-factor for the nuclear Dicer enzyme Drosha in the miRNA pathway, showed an obvious difference in mRNA expression among different tissues (**Figure 1**).

The major finding of this expression study was the very low to absent expression of the mRNA that encodes the small dsRNA-binding protein R2D2 in all silkmoth tissues (**Figure 1**). It is noted that only a partial cDNA clone of *Bombyx R2D2*, corresponding to its 5′-part, was obtained. Moreover, it was also observed that in some amplifications, the PCR product was characterized by a frameshift in the ORF. It is noted that the observed frameshift occurs at the beginning of the second dsRNA-binding domain which results in its deletion. Therefore it is likely that the function of the protein is considerably affected (**Figure S1**). Thus, it appears that in the Daizo strain at least two alleles of *BmR2D2* exist, that they differ in their 5′-parts and of which one allele corresponds with the annotated sequence, while the other one is affected by a deletion. In addition, the 3′-parts of the ORF could not be amplified, using primers based on the annotated sequence. Rapid amplification of cDNA ends (3′-RACE) could be applied to isolate the coding sequence for the C-terminus of BmR2D2 in the Daizo strain. These experiments should determine whether a functional BmR2D2 protein can be encoded by the mRNA. However, it is obvious that the *BmR2D2* mRNA is expressed at very low levels (5′-part is only clearly detectable after 40 cycles of amplification) and therefore represents a limiting factor in the RNAi core machinery. In *Drosophila*, where the action mechanism of the RNAi machinery has been investigated at the molecular level, it was shown that *R2D2* is involved in the loading of siRNAs, the small RNAs generated by Dicer-2, onto Ago-2, which is the central effector in the RISC complex involved in defense against transposable elements and viral infections [17], [20].

Despite the absence of *R2D2* expression, it was observed that Bm5 cells could carry out specific gene silencing after transfection of dsRNA (**Figure 3A**). This indicates that BmR2D2 is not an essential component of the siRNA machinery in silkmoth cells. It is possible that another dsRNA-binding protein, Loquacious (Loqs), which is ubiquitously expressed (**Figure 1**), can compensate for the absence of BmR2D2, as was observed in *Drosophila* [32]. In *Drosophila*, the Dicer-2/R2D2 complex is involved in the processing of siRNAs from exogenous origin (for instance, derived from RNA viruses) while the Dicer-2/Loquacious complex targets siRNAs that are endogenously produced and are derived from transposable elements or transgenes [33]. However, some studies have shown that in S2 cells R2D2 can be dispensable even for triggering silencing by exogenous dsRNAs [20], [34]. Also a functional piRNA pathway was demonstrated in the *Bombyx*-derived BmN4 cell line [35] and the involvement of piRNAs in the silencing of mRNAs was recently demonstrated in *Drosophila* embryos [36]. Although the exact mechanism of dsRNA-mediated gene silencing in *Bombyx* cells remains to be determined, it does not depend on the R2D2 dsRNA-binding protein, when dsRNA is introduced efficiently inside the cells as in case of transfection.

In contrast to transfection, plain addition of dsRNA to the culture medium of Bm5 cells (“dsRNA soaking”) has no gene-silencing effect (**Figure 3B**; see also [22]), which has been also observed in other lepidopteran cell lines [37], [38]. This observation contrasts with that of *Drosophila*-derived S2 cells where efficient RNAi is achieved when cells are “soaked” at doses between 5–20 µg/ml of dsRNA in the extracellular medium [27]. Our studies show that this deficiency in Bm5 cells does not originate from a non-functional core RNAi machinery and therefore could be caused by the inability of the cells to internalize efficiently dsRNAs. However, studies have shown that fluorescently labelled dsRNA can be internalized by lepidopteran cells [11] and it is therefore proposed that a functional barrier exists between internalization (which occurs by endocytosis in S2 cells [20] and presumably in Bm5 cells as well) and presentation to the RNAi machinery in the cytoplasm or RNA-processing GW bodies. Our studies do not exclude a possible role for BmR2D2 in the latter process, for instance by increasing the sensitivity of the RNAi machinery to respond to small amounts of dsRNA that are transported into the cytoplasm, or by an unknown mechanism (for a discussion of the possible failure of TcR2D2 to complement BmR2D2, see further below). The survey of expression levels of components of small RNA pathways also revealed the absence of the mRNA for the full-length BmTranslin (BmTranslin2; **Figure 2**) protein in Bm5 cells, but not in silkmoth tissues (**Figure 1**). On the other hand, the mRNAs of a truncated form of Translin, with a deletion of the conserved C-terminus (BmTranslin1; **Figure 2**), as well as that of the heteromerization partner of Translin, Trax-B, were readily detected in Bm5 cells (**Figure 1**; data not shown). Heterologous expression in Hi5 cells revealed that BmTranslin2 was produced at high levels while BmTranslin1 was barely detectable (**Figure 4** **; Figure S2**). The interaction of Translin and Trax-B proteins to form a functional complex involved in nucleic acid binding is conserved in yeast, *Drosophila* and mammals and this interaction requires the C-terminal domain of Translin [39]. Trax-B protein is stabilized by co-expression of Translin [40] and a functional complex will therefore presumably be formed in the presence of the BmTranslin2 but not the BmTranslin1 isoform. It is also possible that the interaction with BmTrax-B stabilizes BmTranslin2 which could explain the low stability of BmTranslin1 protein.

It is striking that, for both *BmR2D2* and *BmTranslin* genes, mutant, and possibly dysfunctional, alleles were isolated (**Figure 2**). This may reflect the fact that these genes are not essential for *Bombyx* development, as is the case in *Drosophila* [17], [40]. Moreover, the domestication of the silkmoth for thousands of years and the associated protection from its natural hostile environment could have resulted in the loss of genes involved in the immune response against RNA viruses [41], [42] in which *BmR2D2* and possibly also *BmTranslin* may have been involved, at least in some strains. It is clear that some silkmoth strains have a functional *R2D2* gene since a full-length sequence has been deposited in Genbank (accession NM_001195078) but our results indicate that mutant alleles have also accumulated. Since RNAi experiments through injection into the hemolymph have been carried out with variable success in *Bombyx* [11], it will be interesting to check whether the success rate of RNAi is correlated with detectable expression levels of a functional *BmR2D2* allele in the tested silkmoth strains (as well as in other lepidopteran species).

We have tried to stimulate the RNAi efficiency in Bm5 cells in experiments that over-express *Tribolium* R2D2. The gene *TcR2D2* is highly expressed in *Tribolium* tissues (unpublished results) and therefore can be correlated with the high efficiency of RNAi that is observed in this species [10]. However, ectopic expression of *TcR2D2* did not result in stimulation of specific gene silencing in both experiments of transfection or “soaking” with dsRNA (**Figure 3**). As discussed before, RNAi efficiency may depend on other factors than R2D2 in Bm5 cells. On the other hand, it is also possible that *Tribolium* R2D2 is a suboptimal choice to complement for the absence of BmR2D2 in Bm5 cells. At the amino acid-level, sequence identities between *Bombyx* R2D2 and *Tribolium* and *Drosophila* R2D2 are 22% and 16%, respectively, while similarities amount to 30–39% (**Figure 4**). The highest identities/similarities are found in the two dsRNA-binding domains (21–34% and 39–60% respectively). The low conservation is not surprising since the antiviral immunity genes *Dicer-2*, *R2D2* and *Ago-2* are among the fastest evolving of all genes in the genus *Drosophila* [43] and presumably in other insect groups as well. The fast evolution of the genes is triggered by an evolutionary “arms race” between the RNAi-based host defense system and evolving viral RNAi suppressors. Differences could also exist with respect to Translin function between *Bombyx* and *Tribolium* since no homolog of the dimerization partner of Translin, Trax, was found in the *Tribolium* genome (J. Baert, H.H., L.S., and G.S., unpublished results). Because of the high divergence, there is a distinct possibility that TcR2D2 is not capable to form a functional complex with BmDicer-2 and participate in the RNAi process in Bm5 cells. Nevertheless, it is noted that sequence conservation between BmR2D2 and BmLoquacious is also low (23% identity and 39% similarity; data not shown). In *Drosophila* S2 cells Loquacious is capable to enter the siRNA pathway and form complexes with Dicer-2 for the processing of endogenous dsRNAs which are derived from transposable elements [32]. This observation may point to a remarkable flexibility of the small RNA silencing pathways to recruit different types of factors with similar functions. *In vitro* experiments should determine whether BmDicer-2/TcR2D2 dimers can bind siRNAs and nucleate RISC formation as is observed for the DmDicer-2/DmR2D2 complex in purified S2 cell extracts [17]. It would also be valuable to isolate full-length BmR2D2 from appropriate silkmoth strains and test its ability to stimulate RNAi triggered by intracellular (following transfection) or extracellular (in “soaking” experiments) dsRNA.

Immunofluorescence staining of transfected Hi5 cells shows that both TcR2D2 and BmTranslin are located in defined spots in the cytoplasm of the cells. A similar pattern of staining was also observed in Bm5 cells (data not shown), although evidently in a lower number of cells, because of the lower transfectability of the cell line [27]. A diffuse pattern of staining was also observed for BmTranslin2 (**Figure 5B**). It is not likely that the spots represent aggregates, since the protein is preferably found in the soluble fraction (**Figure 5A**). Regarding this defined distribution, a relevant observation may be that a transformed Bm5 cell line that over-expresses TcR2D2, displays cellular extensions that can also be observed when cells are induced to differentiate with 20-hydroxy-ecdysone (data not shown). Moreover, a role in cell differentiation in ovarian follicles was demonstrated for *R2D2* in *Drosophila* [44]. Also Translin has been proposed to function in regulating the expression of a variety of mRNAs, by regulating RNA translocations and localization within cells [39]. RNAi functional assays indicate that, similar to TcR2D2, BmTranslin2 does not stimulate specific gene silencing after transfection of dsRNA in Bm5 cells (data not shown). However, because of their similar confined cellular localization, it is suggested that both RNA-binding proteins function in the same differentiation pathway. It can be hypothesized that, through the use of their RNA-binding domains, these proteins could influence the local translation of mRNAs by direct binding of mRNAs, or by sequestration of miRNAs, that regulate the translation of mRNAs. Because BmTranslin and TcR2D2 accumulate in the soluble fraction of the cytoplasm (**Figure 5A**), they could be purified with relative ease through the use of their C-terminal hexa-His tags with affinity chromatography. Cloning and sequencing of bound RNAs would then provide valuable information regarding the function of these proteins in cellular differentiation.

In summary, the experiments described in this work provide a first approach to the understanding of the efficiency of the RNAi response in the silkmoth and in Lepidoptera in general. Unexpectedly, it was found that R2D2, an essential co-factor of Dicer-2 in *Drosophila* in the response against exogenous dsRNAs, is not expressed in silkmoth tissues. This could possibly point to the presence of a suboptimal RNAi response against exogenous sources of dsRNA, such as RNA viruses, at least in some strains of the silkmoth. While sequences of small RNA libraries from silkmoth tissues have been published, these studies have mostly focused on the presence of miRNAs [45], [46]. A formal demonstration that RNAi in Lepidoptera is working through the generation of siRNAs by a Dicer-2/R2D2 complex has not been published yet. However, our study demonstrates that specific gene silencing occurs in silkmoth cells, if dsRNA is introduced efficiently in the cytoplasm of the cells by transfection. This process of specific gene silencing triggered by intracellular dsRNA occurs despite the absence of expression of *BmR2D2*. Whether BmR2D2 is somehow involved in the systemic RNAi response, during which dsRNA is taken up by the cells and presented to the RNAi machinery, remains to be investigated.

## Materials and Methods

### mRNA expression studies using RT-PCR

Reverse transcription-coupled polymerase chain reaction (RT-PCR) amplification was carried out for following factors of the core machinery of the miRNA pathway: Drosha, Pasha, Dicer-1, Loquacious and Ago-1 [6]; and the siRNA pathway: Dicer-2, R2D2 and Ago-2 [41]. Of these, Drosha/Pasha and Dicer-1/Loquacious correspond respectively to the nuclear and cytoplasmic Dicer complex involved respectively in the generation of pre-miRNAs and its subsequent conversion to mature miRNAs in the cytoplasm. Ago-1 is the Argonaute protein that forms the core of the miRNA effector RISC. Dicer-2, R2D2 and Ago-2 are the paralogs of Dicer-1, Loquacious and Ago-1 in the siRNA pathway [41]. In addition, the presence of the following auxiliary factors was investigated: Hen1, a small RNA methyltransferase which methylates the 3′-ends of piRNAs and siRNAs [47], and the two partners of the C3PO endonuclease, Translin and Trax-B (common to miRNA and siRNA pathways; [18]). Of these genes, Loquacious, Ago-1, Dicer-2, R2D2, Ago-2 and Translin have been annotated in the *Bombyx* genome: respective accession numbers are NM_001195079, AB332314, NM_001193614, NM_001195078, NP_001036995 and NM_001046817. To identify homologs of Drosha, Pasha, Dicer-1, Hen1 and Trax-B, tBLASTn searches were carried out using amino-acid sequences from *Drosophila*, *Tribolium* or *Apis mellifera* as query. In all cases, a single sequence with high sequence identity/similarity (e-value of 10^−18^ or lower) was identified which was used to design primers to detect homologous sequences in *Bombyx*. An overview of all primers used to detect the mRNAs of the above core factors in the small RNA silencing pathways is presented in **Table 1**. RNA extractions and RT-PCR reactions were carried out as described [48]. Larval tissues were dissected from 5^th^ instar larvae at day 4–5 after the molt. Pupal tissues were collected at day 5 after larval-pupal ecdysis. RNA extracts were prepared from tissues derived from at least three animals of the silkmoth strain Daizo.

### Expression and reporter constructs

To generate expression constructs for *Tribolium* R2D2 (TcR2D2) and *Bombyx* Translin (1^st^ and 2^nd^ isoform or BmTranslin1 and BmTranslin2; see below), the corresponding ORFs were amplified by PCR from *Tribolium* whole body, *Bombyx* ovary and central nervous system cDNA samples, respectively, using the primers 5′-ACTT*GGATCC* CAAC **ATG**TCCCGACAAAATACAAAAACC-3′ (forward) and 5′-ACTT*GGATCC*CCCTTTGGCACCCTTGGTCATATAAA-3′ (reverse) for TcR2D2 and 5′-ACTT*GGATCC* CAAC **ATG**TGCGATAATGAATTGATC-3′ (common forward primer) and either 5′-ACTT*GGATCC*TTTTCTCTTCAAGTTCAATAG-3′ for BmTranslin1 or 5′-ACTT*GGATCC*TTTATGTTCGTGTTCTGGTCC-3′ for BmTranslin2 as reverse primers. Both forward primers contain a BamHI cloning site (italic), a Kozak initiation sequence (underlined) and an ATG start codon (bold). The reverse primers contain also a BamHI cloning site (italic) which immediately precedes the reverse complement sequence of the last codon of the ORFs and allows in-frame cloning with the C-terminal Myc-His sequence in the pEA-MycHis lepidopteran expression vector [27]. PCR products were digested with BamHI and cloned in the corresponding site of the pEA-MycHis vector to generate the pEA-TcR2D2-MycHis, pEA-BmTranslin1-MycHis and pEA-BmTranslin2-MycHis expression vectors. The reporter construct, pA-Luc, was constructed by subcloning the firefly luciferase ORF-SV40 poly(A) sequence from the pGL3 vector (Promega) as a HindIII-BamHI fragment downstream of the actin promoter in the pBmA expression plasmid [49]. The ecdysone-inducible reporter construct pERE-gfp has been described before [16].

### Generation of dsRNA

pLitmus 28i vectors (New England Biolabs) containing 0.3–0.7 kb fragments from the ORFs of firefly luciferase, GFP, BmCAP [50] and BmHR3 [51] were linearized by EcoRI or HindIII and subjected to RNA synthesis reactions using T7 RNA polymerase according to the manufacturer's insructions (Fermentas). After digestion of the template DNA with RQ1 RNase-free DNase (Promega), synthesized ssRNA was purified by phenol-chloroform extraction/ethanol precipitation, re-suspended in annealing buffer (150 mM NaCl, 1 mM EDTA) and annealed with its complement by heating at 94°C for 2 min followed by slowly cooling to room temperature.

### Cell lines, Transfections and RNAi functional assays

Bm5 cells [15] or Hi5 cells [52] were transfected according to established protocols [49]. For expression studies, 1.5 µg/ml of pEA-TcR2D2-MycHis, pEA-BmTranslin1-MycHis or pEA-BmTranslin2-MycHis expression vectors together with 1 µg/ml of pBmIE1 helper plasmid encoding the ie-1 gene for *B. mori* nuclear polyhedrosis virus (BmNPV) [53], [54] was used.

For intracellular RNAi experiments, Bm5 cells were transfected with 0.9 µg/ml of pA-Luc, 0.2 µg/ml of pERE-gfp, 0.9 µg/ml of pEA-TcR2D2-MycHis and 1 µg/ml of nucleic acid (DNA, non-specific dsRNA or specific dsRNA at different ratios). Two days after transfection, RH-5992 (Rohm and Haas Co; [56]) or chromafenozide (Sankyo Agro Co. Ltd; [57]) was added at 200 nM to induce the ecdysone reporter gene (used as normalization for luminescence measurements).

In the experiments that evaluated RNAi by addition of dsRNA to the extracellular medium, Bm5 cells were transfected with 1.4 µg/ml of pA-Luc, 0.2 µg/ml of pERE-gfp and 1.4 µg/ml of pEA-PAC (negative control) or pEA-TcR2D2-MycHis. Before transfection, cells were incubated for two days with different concentrations of nucleic acid (DNA, non-specific dsRNA, specific dsRNA) at a concentration of 15 µg/ml. After transfection, cells were incubated with the same concentrations of nucleic acid for another two days. RH-5992 or chromafenozide was subsequently added at a concentration of 200 nM and luminescence or fluorescence in individual wells was quantified 24 hrs later.

Soluble cellular extracts of transfected cell populations were prepared as described above and directly used for fluorescence measurements or processed for luminescence measurements using the Steady-Glo® Luciferase Assay System kit (Promega) according to the manufacturer's instructions. Specific luciferase or fluorescence activities were calculated as luminescence/fluorescence or fluorescence/luminescence ratios. Both fluorescence and luminescence measurents were carried out with an Infinite M200 luminometer (Tecan) ([57]).

### Western blot analysis

Protein gel electrophoresis and Western blot analysis were carried out as described [55]. Transfected cells were collected by centrifugation and pellets were suspended in phosphate-buffered saline (PBS; 100 µl per 10^6^ cells). After freezing for 15 min at −70°C, the cell suspension was subjected to high speed centrifugation (12000 g, 15 min) and both supernatants (as soluble protein fraction) and cell pellets (as insoluble protein fraction) were collected. Supernatants were diluted 1∶1 (v∶v) with cracking buffer while cell pellets were solubilised in 200 µl cracking buffer [50]. Thirty µl of each fraction was loaded in individual lanes of protein gels. The antibodies that were used in Western blot analysis were mouse anti-Myc (Cell Signalling; at 1∶1000) and rat anti-tubulin (Serotec; at 1∶1000). Corresponding secondary HRP-conjugated anti-mouse and anti-rat (both Chemicon) antibodies were used at 1∶1000. Pierce SuperSignal West Pico chemiluminescent substrate (ThermoScientific) was used for detection.

### Immunofluorescence microscopy

Fluorescent staining of Bm5 or Hi5 cells with specific antibodies was carried out as described before [58]. For detection of Myc-tag fusion constructs, primary mouse anti-Myc and secondary FITC-labeled anti-mouse (Sigma) antibodies were both used at 1∶200. The cells were stained with DAPI and finally mounted in Mowiol 4–88 (Sigma) and observed with a wide field Nikon TE 2000 fluorescence microscope.

### Sequence comparison and phylogenetic analysis

Alignment of BmR2D2 (accession NM_001195078), DmR2D2 (AAF52561) and TcR2D2 (NP_01128425) was generated by MegAlign in DNAStar with ClustalW method [59]. Based on the multiple alignment, the phylogenetic tree was constructed and the sequence divergence was calculated.

## Supporting Information

Figure S1 **Additional evidence for very low to absent expression of BmR2D2 in silkmoth tissues and Bm5 cells.** **Panel A:** Amplification of BmR2D2 for 40 cycles using an independent set of cDNAs from tissues and Bm5 cells using primer pair 1. Indicated with asterisk is the product from testis tissue of 414 bp of which the sequence is shown (with deletion in the third exon) in **Panel B**. Weak amplification products are apparent in other reactions but their identities were not determined. **Panel B:** Sequence of BmR2D2 partial cDNA fragment generated by PCR using primer pair 1 that is indicated in **Panel A** by asterisk and the comparison with the Genbank sequence of BmR2D2 mRNA (NM_001195078). Splice sites are indicated by vertical arrows. The 83 bp deletion in the third exon, which is absent in the amplified fragment is indicated, together with the frame-shift in the amino-acid sequence after the deletion. The sequences corresponding to the two dsRNA-binding domains are underlined. **Panel C:** Amplification of BmR2D2 for 35 cycles from tissues and Bm5 cells using primer pair 5. While no products are generated from cDNA samples, a clear amplification product of 196 bp was obtained from 300 ng of genomic DNA (lane “G”). Abbreviations: larval tissues: EP = epidermis; FB = fat body; G = midgut; M = muscle; MT = Malpighian tubules; CNS = central nervous system; SG = silk gland; T = testis; OV = ovary; H = haemocytes; pupal tissues: WD = wing disk; FB = fat body; G = midgut; MT = Malpighian tubules; CNS = central nervous system; T = testis; OV = ovary. The column “-” shows amplifications in the absence of template. MW marker is the 100 bp ladder from Fermentas.
(TIF)

Figure S2 **Comparison of expression levels of BmTranslin2, BmTranslin1 and TcR2D2 in transfected Hi5 cells.** **Left:** Western blot analysis of expression of TcR2D2 and BmTranslin1 (exposure time = 1 hour). Comparison of the intensity of detection signals indicate that expression levels of TcR2D2 exceed those of BmTranslin1 by more than 200-fold. **Right:** Western blot analysis of expression of TcR2D2 and BmTranslin2 (exposure time = 5 minutes). Comparison of the intensity of detection signals indicate that expression levels of TcR2D2 exceed those of BmTranslin1 by more than 5-fold. It is therefore suggested that expression levels between the BmTranslin isoform proteins differ by more than 1,000 fold. Note also the detection of signals of higher and lower MW for BmTranslin2, indicative of formation of dimers and degradation products, respectively. Blots that detect tubulin expression are included as loading controls.
(TIF)

Text S1 **Sequences of PCR products shown in** **Figures 1** **and S1 that correspond to factors of the RNAi machinery.** Indicated are the length of the PCR fragments and the tissue of origin from which they were amplified. Because PCR products of the same length were amplified from the other tissues, it is assumed that the PCR products from the other tissues have the same sequence. In the DNA sequences of the PCR products are indicated the primers (in red) and the locations of the different exons (by absence/presence of underlining). Under the DNA sequence is indicated the corresponding amino acid sequence of the RNAi factor. For the BmLoqs sequence, it is noted the sequence of the PCR product became unreadable in the middle of the fragment; it is assumed that this PCR product corresponds to several different fragments that correspond to mRNA isoforms of *BmLoqs*.
(DOC)
