# Supplementary figures and images for: Search for Limiting Factors in the RNAi Pathway in Silkmoth Tissues and the Bm5 Cell Line: The RNA-Binding Proteins R2D2 and Translin
Source: PLoS One. 2011 May 26;6(5):e20250. doi: 10.1371/journal.pone.0020250 (PMC3102679; doi:10.1371/journal.pone.0020250)

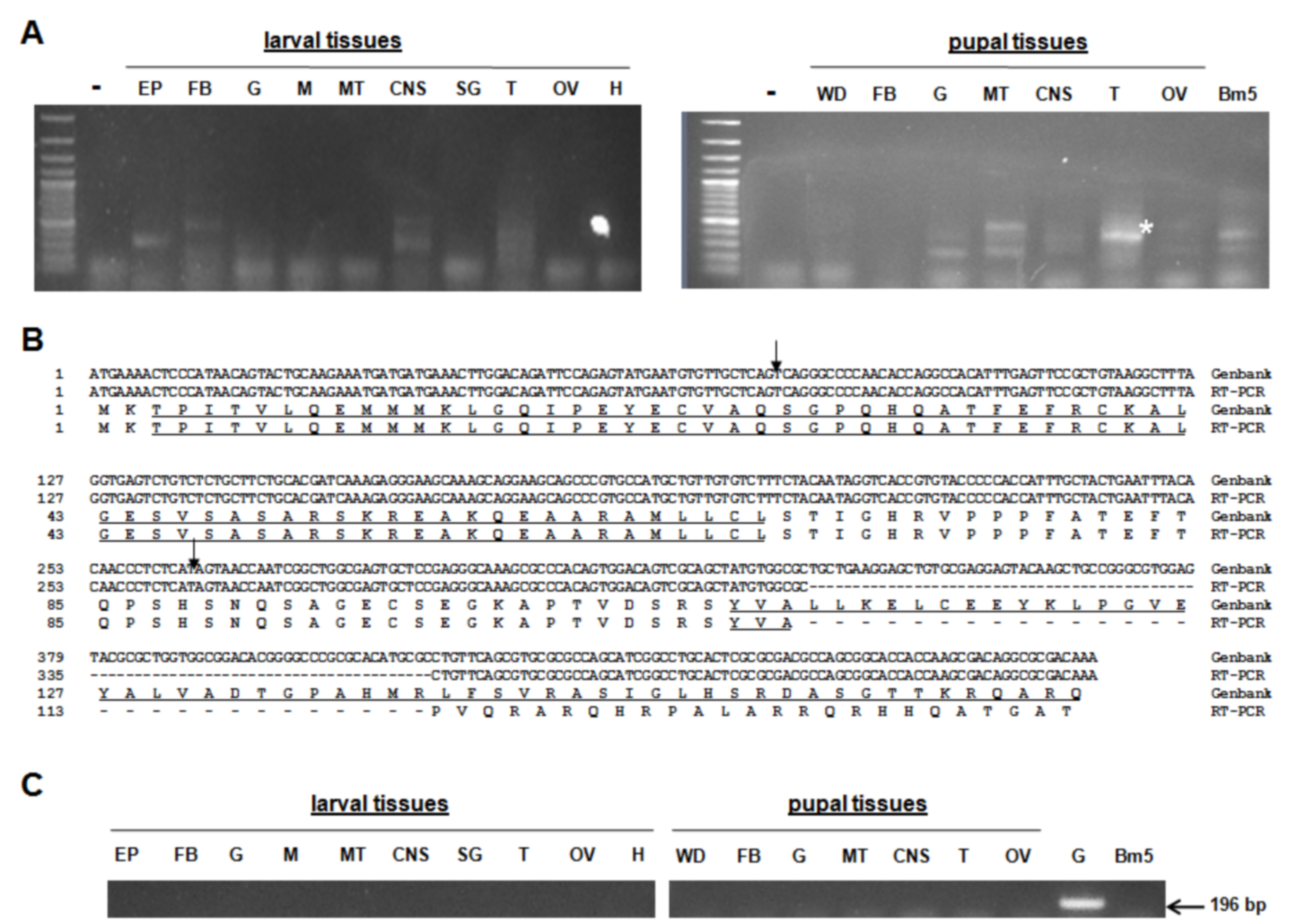

Supplement: Figure S1 — Additional evidence for very low to absent expression of BmR2D2 in silkmoth tissues and Bm5 cells. Panel A: Amplification of BmR2D2 for 40 cycles using an independent set of cDNAs from tissues and Bm5 cells using primer pair 1. Indicated with asterisk is the product from testis tissue of 414 bp of which the sequence is shown (with deletion in the third exon) in Panel B. Weak amplification products are apparent in other reactions but their identities were not determined. Panel B: Sequence of BmR2D2 partial cDNA fragment generated by PCR using primer pair 1 that is indicated in Panel A by asterisk and the comparison with the Genbank sequence of BmR2D2 mRNA (NM_001195078). Splice sites are indicated by vertical arrows. The 83 bp deletion in the third exon, which is absent in the amplified fragment is indicated, together with the frame-shift in the amino-acid sequence after the deletion. The sequences corresponding to the two dsRNA-binding domains are underlined. Panel C: Amplification of BmR2D2 for 35 cycles from tissues and Bm5 cells using primer pair 5. While no products are generated from cDNA samples, a clear amplification product of 196 bp was obtained from 300 ng of genomic DNA (lane “G”). Abbreviations: larval tissues: EP = epidermis; FB = fat body; G = midgut; M = muscle; MT = Malpighian tubules; CNS = central nervous system; SG = silk gland; T = testis; OV = ovary; H = haemocytes; pupal tissues: WD = wing disk; FB = fat body; G = midgut; MT = Malpighian tubules; CNS = central nervous system; T = testis; OV = ovary. The column “-” shows amplifications in the absence of template. MW marker is the 100 bp ladder from Fermentas. (TIF) [file pone.0020250.s001.tif]

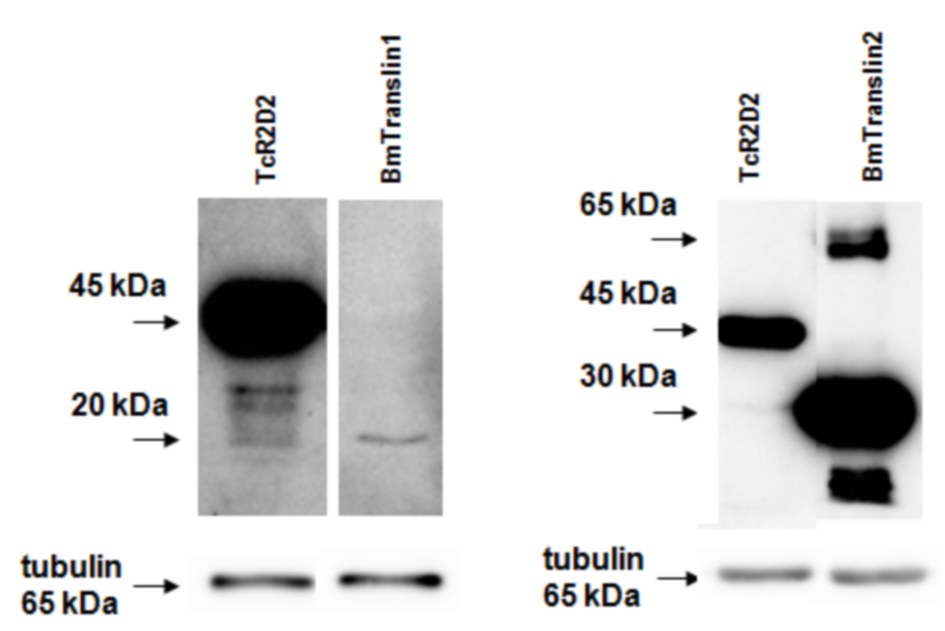

Supplement: Figure S2 — Comparison of expression levels of BmTranslin2, BmTranslin1 and TcR2D2 in transfected Hi5 cells. Left: Western blot analysis of expression of TcR2D2 and BmTranslin1 (exposure time = 1 hour). Comparison of the intensity of detection signals indicate that expression levels of TcR2D2 exceed those of BmTranslin1 by more than 200-fold. Right: Western blot analysis of expression of TcR2D2 and BmTranslin2 (exposure time = 5 minutes). Comparison of the intensity of detection signals indicate that expression levels of TcR2D2 exceed those of BmTranslin1 by more than 5-fold. It is therefore suggested that expression levels between the BmTranslin isoform proteins differ by more than 1,000 fold. Note also the detection of signals of higher and lower MW for BmTranslin2, indicative of formation of dimers and degradation products, respectively. Blots that detect tubulin expression are included as loading controls. (TIF) [file pone.0020250.s002.tif]
