# Supplementary material for: Search for Limiting Factors in the RNAi Pathway in Silkmoth Tissues and the Bm5 Cell Line: The RNA-Binding Proteins R2D2 and Translin
Source: PLoS One. 2011 May 26;6(5):e20250. doi: 10.1371/journal.pone.0020250 (PMC3102679; doi:10.1371/journal.pone.0020250)
Supplement: Text S1 — Sequences of PCR products shown in Figures 1 and S1 that correspond to factors of the RNAi machinery. Indicated are the length of the PCR fragments and the tissue of origin from which they were amplified. Because PCR products of the same length were amplified from the other tissues, it is assumed that the PCR products from the other tissues have the same sequence. In the DNA sequences of the PCR products are indicated the primers (in red) and the locations of the different exons (by absence/presence of underlining). Under the DNA sequence is indicated the corresponding amino acid sequence of the RNAi factor. For the BmLoqs sequence, it is noted the sequence of the PCR product became unreadable in the middle of the fragment; it is assumed that this PCR product corresponds to several different fragments that correspond to mRNA isoforms of BmLoqs. (DOC) [file pone.0020250.s003.doc]

**Sequenced PCR Fragments**

**Primers are indicated in red at the ends of the DNA sequences.**

**Different exons are indicated by absence/presence of underlining.**

**Indicated is also the corresponding amino-acid sequence after conceptual translation.**

**BmDrosha fragment (357 bp)**

**(from larval brain)**

**CGTTCACGGATCGCTCAGTCGGCTTCACTCTCCTCACGCTCGGATCCAACCAGCGCTTAGAGTTCCTCGGCGACACGGTGCTCCAGCTGGTCGTGTCCGATCGACTGTACAGACACTTCCCTGATCATCACGAAGGCCATTTGTCGCTGTTACGGTCGTCTCTGGTCAACAACAGAACACAGTCTATGGTTTGCGACGATCTGAACATGTCTGCGTACGCCATATACAATAATCCTAAAGCGAAACCGACCACAAAGAAACATAAGGCTGATTTACTCGAGGCTTTCTTGGGAGCATTGTTTATTGATAAGAACCTGGAGTACTGTCAGGCGTTCTGCAACGCGTGTCTGTTTCCGC**

**(frame 3)**

**FTDRSVGFTLLTLGSNQRLEFLGDTVLQLVVSDRLYRHFPDHHEGHLSLLRSSLVNNRTQSMVCDDLNMSAYAIYNNPKAKPTTKKHKADLLEAFLGALFIDKNLEYCQAFCNACLFP**

**BmPasha fragment (404 bp)**

**(from larval testis)**

**GTCAGCACCAGCTGGAAGACCCAGCACTTGACTCACGAGCAGGTCAACGAGTACTGCGCTAAGCTGTTCAAGTTTAAGACCGTCAATATTATGCTTTTCAAGAGGCGCTGGGCCGACAAGCGCAAGTACACCAAGGCGCGGAAGGCCCTGCAGTACCCGGCGCTGCCCGAGGGCACCAAGCTCATCGCCATCCCCCTAACGCTGCCACTGACGGCTGCATTGCGAGCAGGCGGCAAGTCCACCAAGCGCGACTGGGTCATGAACATGGACGGGCGCAGCTACCTCGCCGTGTTCCACGAGTACGTGGGCCGCGCCCTGCAGAAGCAGCCCGTCTACGAGTTCAAGCAGCTGGGAGAACGCCGCCACGCCCTACCAGGCCACGGTGTACATCGGCGGCATGCAGT**

**(frame 1)**

**VSTSWKTQHLTHEQVNEYCAKLFKFKTVNIMLFKRRWADKRKYTKARKALQYPALPEGTKLIAIPLTLPLTAALRAGGKSTKRDWVMNMDGRSYLAVFHEYVGRALQKQPVYEFKQLGERRHALPGHGVHRRHA**

**BmDicer-1 fragment (266 bp)**

**(from pupal testis)**

**TGAAGCCGGGTGAGGTGTTCGTGTTTGACCCCGACAAGTACAAGGAGGCGGTCGTGACGCCGTGGTACAGGAACCAGGACCAGCCGCAGTACTTCCTGGTGGCGGAGATATGCTGGCGCCTGAGTCCCGACTCCGTGTTCCCGTCAGCGAGCCACGCGACCTTCCGCGACTACTACCAGAACAAGTACGGCGTCACCATCACGCAGAGCAAGCAGCCGCTGCTGGACGTGGACCACACCAGCGCCAGACTCAACCTCCTCACTCCC**

**(frame 3)**

**KPGEVFVFDPDKYKEAVVTPWYRNQDQPQYFLVAEICWRLSPDSVFPSASHATFRDYYQNKYGVTITQSKQPLLDVDHTSARLNLLTP**

**BmLOQS fragment (> 495 bp)**

**(from larval ovary)**

**GAGCTGTTGGCACGTCGTGGAACTGTTCCTAAATATGAACTAGTCCAGATAGAAGGCATGATACATGAACCCACTTTCCGGTACAGAGTAACTGTGGCTGATTTAGTTGCAATGGGCACTGGTCGCTCAAAAAAAGAGGCAAAGCACTCTGCAGCCAAGGCTTTACTGGACAAGTTAACTGGTGCTACACCAGCTGATCAGACTACCAATGGCAATGTTCCTGAAACTGGTGCTGTAGTACCTACATTTGAAGATAAACTAATGGGTAATCCTGTTGGATGGCTTCAGGAGTTGTGTATGTCACGATTCTGGCCACCACCATCTTACCATGCTGAAAACGATGACAATGTTAATAGAC (heterogenous junction)**

**TTTCAGGTTTGCCCCATGAACGTCATTTTACGATCATTTGCACATTGCTCAAGCGCCGTGAAGTTGGTACAGGCAAGTCAAAGAAATTAGCTAAACGGCAAGCTGCCTACAAGATGTGGCAGGCCTTACAGGACAAC**

**(frame 1)**

**MIHEPTFRYRVTVADLVAMGTGRSKKEAKHSAAKALLDKLTGATPADQTTNGNVPETGAVVPTFEDKLMGNPVGWLQELCMSRFWPPPSYHAENDDNVNR …**

**(frame 3)**

**SGLPHERHFTIICTLLKRREVGTGKSKKLAKRQAAYKMWQALQDN**

**BmAgo-1 fragment (444 bp)**

**(from larval ovary)**

**GGGCGATAGCATGTTTCGCGCCACAAAGAACAGTACGAGAGGATGCTCTCAAGAATTTTACTCAACAACTTCAAAAGATATCCAACGACGCTGGCATGCCAATAATAGGGCAACCCTGTTTCTGCAAGTACGCTACAGGGCCAGACCAAGTGGAGCCTATGTTTAAATACTTAAAGTCTACATTTGTGCAGCTACAGCTCGTTGTCGTCGTGTTACCAGGAAAAACACCTGTCTATGCCGAAGTAAAAAGAGTTGGTGACACAGTATTGGGAATGGCAACTCAATGTGTACAAGCGAAGAACGTAAACAAAACTTCACCGCAGACCCTCAGTAATTTATGTTTAAAAATTAATGTTAAACTGGGAGGCATTAATTCTATTCTCGTTCCATCTCTTCGTCCGAAGGTGTTCAACGAGCCCGTGATCTTCCTGGGCGTGGACGTGA**

**(frame 3)**

**AIACFAPQRTVREDALKNFTQQLQKISNDAGMPIIGQPCFCKYATGPDQVEPMFKYLKSTFVQLQLVVVVLPGKTPVYAEVKRVGDTVLGMATQCVQAKNVNKTSPQTLSNLCLKINVKLGGINSILVPSLRPKVFNEPVIFLGVDV**

**BmDicer-2 fragment (449 bp)**

**(from larval testis)**

**Catacagttcaccgaagaggaagtaatgaccggtgaattaagtgaagaaagctgggaatctgtgatgaataatttccaaaatggcatcgctgaggtagagcccgagggctgtgcccaaaattctatgcaatgctatgtacattcacaagcagtggcggacaaatctatagccgattccgtggaagctctgatcggcacatatctacttagtggagggatattagcagctgtaaaactacttgaatggatggaggtgtttccgccacaggataactttgcagacatgctccataagccggtgcagacaccgttatcgaagaatttagctaccgaagctgatattgactttttactaaacaattcgagagctgatgtcgaaaagatcctaaactacacgttcaaagattcgaccttcctgcttaacgctctgtctcactcgtcgtacatcc**

**(frame 2)**

**IQFTEEEVMTGELSEESWESVMNNFQNGIAEVEPEGCAQNSMQCYVHSQAVADKSIADSVEALIGTYLLSGGILAAVKLLEWMEVFPPQDNFADMLHKPVQTPLSKNLATEADIDFLLNNSRADVEKILNYTFKDSTFLLNALSHSSYI**

**BmR2D2 fragment (497 bp)**

**primer pair 1 ; template: testis cDNA**

***CAAG*ATGAAAACTCCCATAACAGTACTGCAAGAAATGATGATGAAACTTGGACAGATTCCAGAGTATGAATGTGTTGCTCAGTCAGGGCCCCAACACCAGGCCACATTTGAGTTCCGCTGTAAGGCTTTAGGTGAGTCTGTCTCTGCTTCTGCACGATCAAAGAGGGAAGCAAAGCAGGAAGCAGCCCGTGCCATGCTGTTGTGTCTTTCTACAATAGGTCACCGTGTACCCCCACCATTTGCTACTGAATTTACACAACCCTCTCATAGTAACCAATCGGCTGG**

**CGAGTGCTCCGAGGGCAAAGCGCCCACAGTGGACAGTCGCAGCTATGTGGCGCTGCTGAAGGAGCTGTGCGAGGAGTACAAGCTGCCGGGCGTGGAGTACGCGCTGGTGGCGGACACGGGGCCCGCGCACATGCGCCTGTTCAGCGTGCGCGCCAGCATCGGCCTGCACTCGCGCGACGCCAGCGGCACCACCAAGCGACAGGCGCGACAAA**

**translation starting at first ATG:**

**MKTPITVLQEMMMKLGQIPEYECVAQSGPQHQATFEFRCKALGESVSASARSKREAKQEAARAMLLCLSTIGHRVPPPFATEFTQPSHSNQSAGECSEGKAPTVDSRSYVALLKELCEEYKLPGVEYALVADTGPAHMRLFSVRASIGLHSRDASGTTKRQARQ**

**BmR2D2 fragment (414 bp)**

**primer pair 1 ; template: testis cDNA**

***CAAG*ATGAAAACTCCCATAACAGTACTGCAAGAAATGATGATGAAACTTGGACAGATTCCAGAGTATGAATGTGTTGCTCAGTCAGGGCCCCAACACCAGGCCACATTTGAGTTCCGCTGTAAGGCTTTAGGTGAGTCTGTCTCTGCTTCTGCACGATCAAAGAGGGAAGCAAAGCAGGAAGCAGCCCGTGCCATGCTGTTGTGTCTTTCTACAATAGGTCACCGTGTACCCCCACCATTTGCTACTGAATTTACACAACCCTCTCATAGTAACCAATCGGCTGGCGAGTGCTCCGAGGGCAAAGCGCCCACAGTGGACAGTCGCAGCTATGTGGCGCCTGTTCAGCGTGCGCGCCAGCATCGGCCTGCACTCGCGCGACGCCAGCGGCACCACCAAGCGACAGGCGCGACAAA**

**translation starting at first ATG:**

**MKTPITVLQEMMMKLGQIPEYECVAQSGPQHQATFEFRCKALGESVSASARSKREAKQEAARAMLLCLSTIGHRVPPPFATEFTQPSHSNQSAGECSEGKAPTVDSRSYVAPVQRARQHRPALARRQRHHQATGAT**

**BmR2D2 fragment (196 bp)**

**primer pair 5 ; template: genomic DNA**

**AAAGCGCCCACAGTGGACAGTCGCAGCTATGTGGCGCTGCTGAAGGAGCTGTGCGAGGAGTACAAGCTGCCGGGCGTGGAGTACGCGCTGGTGGCGGACACTGGGCCCGCGCACATGCGCCTGTTCAGCGTGCGCGCCAGCATCGGCCTGCACTCGCGCGACGCCAGCGGCACCACCAAGCGACAGGCGCGACAAA**

**(frame 1)**

**KAPTVDSRSYVALLKELCEEYKLPGVEYALVADTGPAHMRLFSVRASIGLHSRDASGTTKRQARQ**

**BmAgo-2 fragment (370 bp)**

**(from larval testis)**

**tctccgattgacttgggcgacgggctcgagatgtggactggtttattccaatctgctatatttacatcgaaagccttcatcaacgttgatgttgcacacaaaggtttcccaaagaatcaacctatgattgatgctttcacgcgtgattttcgtttggaccccaatcgtccagtggatcggcaaccaggccgtgctgctgaagctttcaatgaattcattagagggcttaaggtcgtatcgaagatacttggtaccggaccttcttcgggacaattacgcgagcacatatgcaacggggttgtcgacccaccgtcacgtcaaacatttacgttggagaacgacaaaggaccgccggttaggatgaccgtat**

**(frame 1)**

**SPIDLGDGLEMWTGLFQSAIFTSKAFINVDVAHKGFPKNQPMIDAFTRDFRLDPNRPVDRQPGRAAEAFNEFIRGLKVVSKILGTGPSSGQLREHICNGVVDPPSRQTFTLENDKGPPVRMTV**

**BmHEN1 fragment (475 bp)**

**(from larval ovary)**

**CCCACCAATGTATGTACAGCGATATGCTGCTATTGTCGACTGTCTACTGGACGAACGGTGGAGCGGAAAATTGGATAAGGTGGTAGACCTTGGTTACCATGATATGAGTTTCATTAAGTACCTGAAGGAAGTTTCCGGAATCAAGTCAATTTTGGGAGTTGATCTTGAGACTATACCATTGCAGTGCTCATCAGACTTGTTGAGTTGTAATGAGTATGCACCTAAAAGAGAAACACCATTACAAATTTCACTGCTCCAAGGCAATGCAGCAGACCCAGATTACAGACTTATTGGTTGTGATGCAGTCATAGCCATAGAAATGATAGAACATATGCTACCTCATGACCTGGAACGACTTGTACACACTGTGTTTGCATTTATCAAACCATGGATTGTCATTTTTACCACACCAAATGGTGATTTCAATGTACTGTTCAAATCATTAGAAAAAAATGGCTTGAGAAGGCTCGATCAT**

**(frame 2)**

**PPMYVQRYAAIVDCLLDERWSGKLDKVVDLGYHDMSFIKYLKEVSGIKSILGVDLETIPLQCSSDLLSCNEYAPKRETPLQISLLQGNAADPDYRLIGCDAVIAIEMIEHMLPHDLERLVHTVFAFIKPWIVIFTTPNGDFNVLFKSLEKNGLRRLDH**

**BmTranslin fragment (397 bp)**

**(from larval testis)**

**AGGTATCAGGATCACTGGAGATTCATGACCCAACGCTACTGTTATCTGATAGCGCTCACTATATGGCTAGAGAAGGGAATCCTGGCTTCACACGAGACCATGGCTGAAATATTAGGTGTTAGTCCAGTGGAGTTAAAAGAAGGTTTCCATTTGGACATTGAAGACTATCTGATTGGGCTATTGACGATGTGCTCAGAATTGTCTCGTCTGGCCGTGAACTCGGTGACCCGCGGCGACTACGAGCGCCCCCTGAGGATCTCCAAGTTCGTGATGGAACTGAACGCCGGCTTCAGGCTATTGAACTTGAAGAACGATCATTTGCGCAAACGCTTCGACGCCCTAAAGTACGACGTGAAGAAAATAGAGGAAGTCGTCTTCGTCTACGATCTCAGCATCA**

**(frame 1)**

**RYQDHWRFMTQRYCYLIALTIWLEKGILASHETMAEILGVSPVELKEGFHLDIEDYLIGLLTMCSELSRLAVNSVTRGDYERPLRISKFVMELNAGFRLLNLKNDHLRKRFDALKYDVKKIEEVVFVYDLSI**

**BmTrax-B fragment (362 bp)**

**(from larval epidermis)**

**ACAGTCCGATCTTAGCCATGTTTAAAAATGCTGCTCTAAAACTAAATGAACGACAGGATAGACACGAAAGACTGGTCAAACTCTCGAGAGACATTACTATTGAGAGCAAGAGAATTATTTTCTTGTTACACTCGGCTATTACAGAAGAATCTACAGCAAAAGTAATAGAAGAAGCTAAAGAAAGATTTCAGAAATTAATAAAAGGACCAATTAAATCTGTAGGCTTGGAACTTGAGAACAGCCCTGCATATTTACATTCACGGGCAGTAACTGCAGGTTTCCAAGAATACATTGAAGCAAGAACTTTATTTTCTCTTATGGAGACCAAAAAGCTAATAAGTTGGCCAGAGATACGAGATGAA**

**(frame 3)**

**SPILAMFKNAALKLNERQDRHERLVKLSRDITIESKRIIFLLHSAITEESTAKVIEEAKERFQKLIKGPIKSVGLELENSPAYLHSRAVTAGFQEYIEARTLFSLMETKKLISWPEIRDE**
